# Attenuated *Listeria monocytogenes* as a Vaccine Vector for the Delivery of OMPW, the Outer Membrane Protein of *Aeromonas hydrophila*

**DOI:** 10.3389/fmicb.2020.00070

**Published:** 2020-02-21

**Authors:** Haijuan Zeng, Manman Xie, Chengchao Ding, Junfei Ma, Dongpo Xu, Xiang Wang, Jingxuan Qiu, Qing Liu

**Affiliations:** ^1^School of Medical Instrument and Food Engineering, University of Shanghai for Science and Technology, Shanghai, China; ^2^The Biotechnology Research Institute, Shanghai Academy of Agricultural Sciences, Shanghai, China; ^3^Laboratory for Marine Fisheries Science and Food Production Processes, Qingdao National Laboratory for Marine Science and Technology, Shandong, China

**Keywords:** *Listeria monocytogenes*, attenuation, live vector vaccine, *Aeromonas hydrophila*, safety

## Abstract

*Listeria monocytogenes* (LM) is a gram-positive facultative intracellular pathogen that could stimulate host to produce inflammatory response, cell-mediated immunity, and humoral immunity. In this study, an attenuated live vector vaccine for *Aeromonas hydrophila* (AH) named *EGDeABdd-dat-ompW* was successfully constructed using an attenuated vector named *EGDeABdd*, in which *dal*, *dat*, *act*A, and *inl*B genes were deleted from wild-type LM*-EGDe*. To construct *EGDeABdd-dat-ompW*, a recombinant plasmid pERL3-dat-ompW obtained by inserting the *dat* gene from *EGDe* and outer membrane protein gene *omp*W from AH into pERL3 plasmid was transformed into *EGDeABdd* cell. The safety and immunogenicity of *EGDeABdd-dat-ompW* as an attenuated vector vaccine for delivery of OMPW were assessed through analyzing invasion to Caco-2 cells and mice, cytokine production of macrophagocyte and mouse splenocytes, and T-cell proliferation of mouse splenocytes. Serum titers against AH and the immunoprotective effect of the vaccine to mice were also measured after intravenous injection with vaccine for four times. The results showed that the live vector vaccine *EGDeABdd-dat-ompW* for AH exhibited high attenuation in invading Caco-2 cells and mice than did *EGDe*. Real-time PCR (RT-PCR) showed that cytokines (e.g., TNF-α, IL-6, and IL-1β from macrophages; and IL-6 and IFN-γ from mouse splenocytes) had significantly increased after immunization by *EGDeABdd-dat-ompW*. Meanwhile, the vaccine could induce the production of CD3^+^CD4^+^ and CD3^+^CD8^+^ T-cell proliferation of mice and generate effective immunoprotection against lethal challenge of 20 × LD_50_ AH. All these results indicated that the attenuated *EGDeABdd-dat* could be used as a live vector for the delivery of the exogenous gene, not only possessing safety but also providing high immunogenicity. The successful application in the AH vaccine further showed that it could be used in other fields such as vaccines in cancer or infectious diseases.

## Introduction

*Listeria monocytogenes* (LM), a gram-positive facultative intracellular pathogen, usually causes listeriosis. Upon invading a host cell, LM could escape from the phagocytic cells such as macrophages and dendritic cells (Alberti-Segui et al., 2007) by producing a pore-forming protein, listeriolysin O (LLO), which lyses the vesicular membrane, allowing LM to enter the cytoplasm, where the secreted proteins by LM are degraded to peptides accessed to major histocompatibility complex (MHC) class I molecules of antigen processing and presentation to CD8^+^ T cells. The majority of engulfed bacteria are killed by phagolysosomes, and the degraded proteins access directly to MHC class II molecules of antigen presentation to CD4^+^ T cells (Chen et al., 2014). Meanwhile, LM enhances the antigen presentation effect through the binding of the bacterial wall surface protein and toll-like receptors to antigen-presenting cells. Based on the unique properties mentioned above, it is a promising candidate as a live vector for virus, bacterial disease, or cancer vaccine (Wood and Paterson, 2014).

*Aeromonas hydrophila* (AH) is widely distributed in various water bodies in nature, which is a typical human–animal–fish comorbid pathogen (Janda and Abbott, 2010). AH could produce highly toxic exotoxins, such as hemolysin, tissue toxins, necrotic toxins, enterotoxins, and proteases, which are responsible for diseases such as hemorrhagic septicemia, dropsy, ulceration, asymptomatic septicemia, and exophthalmos, resulting in high mortality in infected animals and huge economic losses in warm water aquaculture. The preferred way is the use of antibiotic to control the disease in aquaculture, which is presently not recommended owing to the risk involved in the development of resistance in pathogens and the transfer of resistance genes to other animals and human pathogens (Karunasagar et al., 1994). Hence, there is an urgent need to use preventative measures. Vaccination is considered as an alternative strategy to protect fish or animals against diseases. However, one of the major problems in the development of vaccine is how to select an effective antigen.

The bacterial outer membrane proteins (OMPs) play a key role in the virulence of the bacteria and are considered potential vaccine candidates (Maiti et al., 2012). The gene (*omp*W) of outer membrane protein W is widely present in gram-negative bacteria, such as *Vibrio* spp. (Jalajakumari and Manning, 1990), *Escherichia coli* (Li et al., 2016), *Salmonella typhimurium* (Morales et al., 2012), *Aeromonas* spp. (Liu et al., 2011), and *Cronobacter sakazakii* (Ye et al., 2018). It is relatively conservative and has good immunogenicity (Maiti et al., 2010), which is inferred to have the cross-immunoprotective effect.

Because of its the strong ability to survive in wide pH and temperature (Chen et al., 2016) and its potential damage to intestinal epithelial cells and the brain (Salazar et al., 2018), LM used for vector should be attenuated in virulence for safety consideration. In this study, attenuated LM named *EGDeABdd*, in which the *act*A, *inl*B, *dal*, and *dat* genes were deleted from wild-type *EGDe*, was used as the vaccine vector. A recombinant plasmid pERL3-dat-ompW was constructed by cloning the *dat* and *omp*W genes into *Sma*I/*Sal*I and *Sal*I/*Sac*I sites of the pERL3 plasmid, and a LLO promoter and secreted signal peptide sequences were cloned in the upstream of the *omp*W gene. The nucleic acid vaccine for AH named *EGDeABdd-dat-ompW* was successfully constructed by electroporation of the recombinant plasmid pERL3-dat-ompW into *EGDeABdd*. The safety and immunogenicity of the *EGDeABdd-dat-ompW* as a vaccine was analyzed.

## Materials and Methods

### Materials

Attenuated strain *EGDeABdd* based on wild-type LM genetic background *EGDe* (BAA-679, serotype 1/2a) was constructed in our laboratory. Wild-type *Aeromonas hydrophila* (CPBC-Ah-0003) was a gift from Dr. Xincang Li (East China Sea Fisheries Research Institute). Plasmid pERL3 was a gift from Professor Qin Luo (Huazhong University of Science and Technology). Primers for gene amplification were synthesized by Sangon (Shanghai, China) listed in Table 1. Six- to 8-week-old Balb/c (in safety evaluation assay) and C57BL/6 mice (in immunity evaluation assay) were purchased from JieSijie (Shanghai, China). All animal studies have been approved by China Ethics Committee and performed in accordance with the ethical standards. Macrophage cell lines Raw264.7 were purchased from Shanghai Cell Bank (Shanghai, China).

**TABLE 1:** Primers used for construction the recombinant plasmid.

Name	Primer sequences	Size (bp)
T1	CCCACCCCGGAATTCCCGGGGTAT AATTGAAAAAATTAACT	978
T2	AAGCTTGGCTGCAGGTCGACGTT ATTTTGCAAACACTAATT	
T3	AGCTCGTGAAGTAC CTAGAAACGAG	440
T4	GTTTTCCGTCAGC TACTTGAACTC	
L1	ATTCTAGACTCGAGAGCTCTAC ATCCATTGTTTTGTAGTT	595
L2	AGAGGAAGGATCTTTTT CATGTAATCCAATCCTTGTATAT	
W1	ATATACAAGGATTGGA TTACATGAAAAAGATCCTTCCTCT	612
W2	CGATTCTAGACTCGAGAGCTCTCTCA GTGGTGGTGGTGGTG GTGGAAGCGATAGCCGACACCAA	

Fluorescence-conjugated anti-mouse antibodies CD3-PerCP-Cy5.5, CD4-FITC, and CD8-PE were from Becton Dickinson (Franklin Lakes, NJ, United States). Brain heart infusion (BHI) medium was purchased from Land Bridge (Beijing, China). Real-time PCR (RT-PCR)-related reagents and rapid cloning kit were from TaKaRa (Dalian, China). PCR and RT-PCR thermal cycler were from Applied Biosystems (United States). Electroporation instrument was from Bio-Rad (United States). Other chemicals were of analytical reagent grade.

### Strain Construction

#### Construction of *EGDeABdd-t* Strain

**FIGURE 1:**
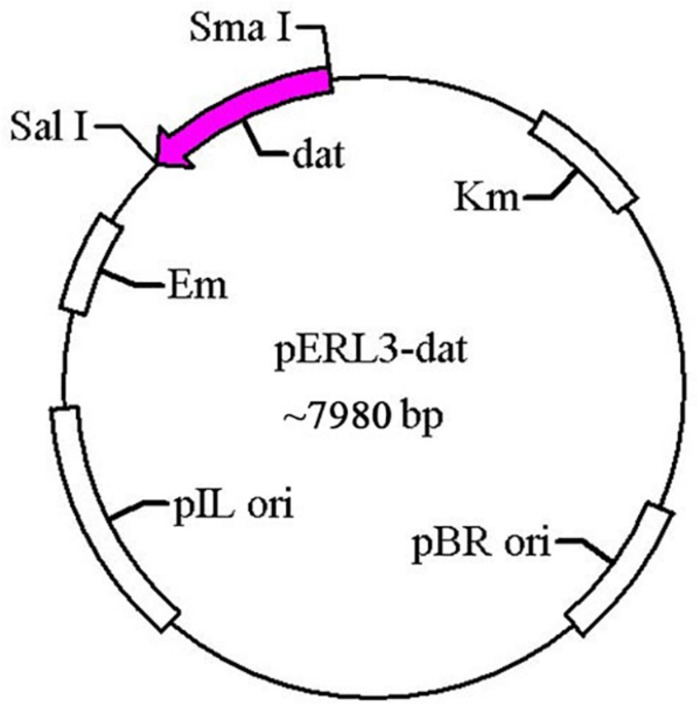
Schematic diagram of recombinant plasmid pERL3-dat. The gene *dat* from *EGDe* was amplified using primers T1 and T2 and inserted at *Sma*I/*Sal*I sites.

The gene *dat* of LM was amplified by PCR using the primers T1 and T2 and cloned into pERL3 at the *Sma*I and *Sal*I sites as shown in Figure 1. The plasmid named pERL3-dat was electroporated into *EGDeABdd* to construct the strain named *EGDeABdd-t*. Positive clones were picked from BHI agar plates and identified by PCR using the primers T3 and T4.

#### Growth Kinetics of *EGDeABdd-t*

To study the growth kinetics of the attenuated strain *EGDeABdd-t* in the absence of D-alanine, the mutant cultured in the presence of 100 μg/ml of D-alanine was washed to remove extracellular D-alanine and suspended in BHI medium without D-alanine. Optical density values (OD values) of bacteria at 600 nm were measured per hour using microplate reader (SpectraMax M2; Molecular Devices, Sunnyvale, CA, United States).

#### Construction of *EGDeABdd-dat-ompW* Strain

**FIGURE 2:**
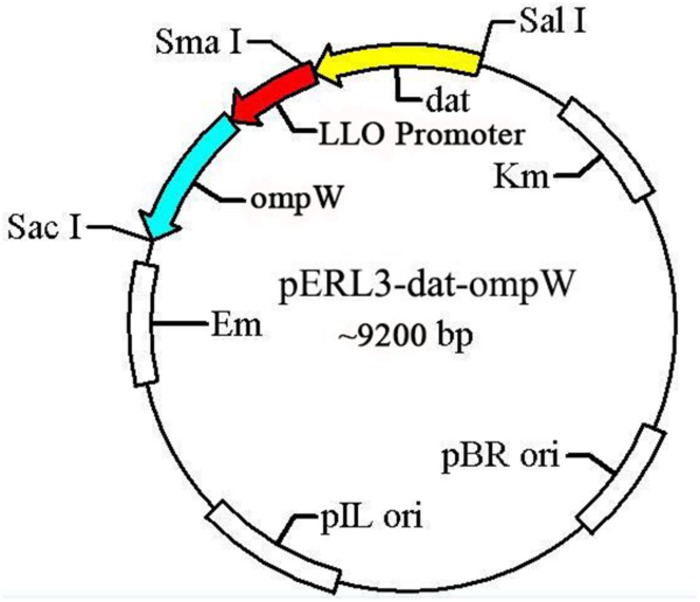
Schematic diagram of recombinant plasmid pERL3-dat-ompW. Listeriolysin O (LLO) promoter sequences of *hly* and *omp*W of AH were amplified using primers L1/L2 and W1/W2, respectively. Two fragments were linked using overlap extension PCR, following by insertion at *Sal*I and *Sac*I sites.

The gene *omp*W of AH and LLO promoter (truncated LLO, containing 45 amino acids) sequences amplified by PCR using the W1/W2 and L1/L2 primers were spliced by overlap extension PCR, following by insertion at of the *Sal*I*/Sac*I sites of recombinant plasmid pERL3-dat as shown in Figure 2. The new recombinant plasmid named pERL3-dat-ompW was electroporated into *EGDeABdd* to construct a vaccine strain named *EGDeABdd-dat-ompW*. Clones identified as positive by PCR using primers W1 and W2 were also determined by sequence analysis.

#### Analysis of Protein Secreted by *EGDeABdd-dat-ompW*

The expression and secretion of the truncated LLO and OMPW (tLLO-OMPW, containing 204 amino acids of OMPW, 45 amino acids of tLLO, and 6 histidine tag) fusion protein was confirmed in the culture supernatants by western blot using anti-His label antibody according to the related literatures. *EGDeABdd-dat* was the control, which was constructed from *EGDeABdd* containing recombinant plasmid pERL3-dat-tLLO.

#### Gene Expression of Strain Detection in Transcription Level

To analyze the difference of the constructs in gene expression, 12 virulence genes and 2 genes related to D-alanine synthesis were analyzed by RT-PCR. Five milliliters of saturated bacterial solution was used to extract RNA. According to kit instructions, the RNA was reverse transcribed into cDNA. RT-PCR was performed using cDNA as a template. The data were processed by 2^–ΔΔ^ ^Ct^ method for relative quantitative analysis, with the gene expression level of *EGDe* as a control and 16S rRNA as an internal reference. The calculation formula is as follows. ΔCt = Ct mean value of strain target gene – Ct mean value of corresponding 16S rRNA, and ΔΔCt = ΔCt of mutant target gene −ΔCt of the *EGDe* corresponding target gene. 2^–ΔΔ^ ^Ct^ is the relative expression level of mutant virulence gene to *EGDe*.

### Safety Assessment

#### *In vitro* Infection to Caco-2 Cells

The human Caco-2 cell lines were used to evaluate the invasiveness of vaccine strains. Cells cultured in a 12-well plates at 10^5^ cells/well were infected by *EGDe*, *EGDeAB-dat*, and *EGDeABdd-dat-ompW* at multiplicity of infection (MOI) = 1:100. After incubation at 37°C for 2 h, the free or adherent bacteria were removed and killed by phosphate-buffered saline (PBS) washing for two times following by 200 μg/ml of penicillin incubation at 37°C for 30 min. The infected cells were lysed in 1% Triton X-100 (Sigma, St. Louis, MO, United States) for 15 min and then gradient diluted to culture in BHI plates containing 200 μg/ml of D-alanine and 5 μg/ml of erythromycin. Colony-forming units (CFUs) were counted 24 h later.

#### The Ability-Induced Caco-2 Cell Apoptosis

After co-culture with *EGDe*, *EGDeAB-dat*, and *EGDeABdd-dat-ompW* for 3 h, Caco-2 cells were harvested and stained with propidium iodide (PI) staining buffer to identify late apoptotic and necrotic cells. The cells were also stained with fluorescein isothiocyanate (FITC)-conjugated annexin V for 15 min to evaluate early apoptosis. Cells of different periods were then analyzed using flow cytometry (Becton Dickinson, Franklin Lakes, NJ, United States).

#### *In vivo* Infection to Mice

A dose of 2 × 10^7^ CFUs *EGDe*, *EGDeAB-dat*, and *EGDeABdd-dat-ompW* were intravenously injected into 6- to 8-week-old Balb/c mice (*n* = 3). On the first day after injection, the spleen and liver were collected from the mice. The single-cell suspension was serially diluted and cultured on chromogenic agar plates. The CFUs per gram for organs were calculated to determine the reduced virulence of a vaccine to mice.

#### Mouse Liver Slices

Three days after intravenous injection of *EGDeABdd-dat-ompW* with a dose of 5 × 10^7^ CFUs (0.1 × 50% lethal dose, LD_50_ about 5 × 10^8^ CFUs), the livers were collected and fixed in formalin for 24 h followed by paraffin embedding, slicing, and hematoxylin and eosin (H&E) staining. Histological morphology of the livers was observed under the microscope. PBS with equal volume was the control.

### Immunological Evaluation

#### Cytokine Measurement

Infections by pathogenic microorganisms elicit host immune responses, especially including inflammation, as innate immunity is responsible typically for initial infection-directed responses. After co-culture at MOI = 100:1 in the presence of *EGDeABdd-dat* or *EGDeABdd-dat-ompW* for 3 h, macrophages Raw264.7 were harvested. RNA was extracted, and RT-PCR was performed to analyze the relative expression of cytokines. The C57BL/6 mice were intravenously immunized with *EGDeABdd-dat* or *EGDeABdd-dat-ompW* of 5 × 10^7^ CFUs (0.1 × LD_50_, LD_50_ about 5 × 10^8^ CFUs), and PBS was the control. On the first day after immunization, mouse spleens were collected, and the RNA was extracted. After reverse transcription of RNA to cDNA, RT-PCR was carried out. The cytokine expression in PBS group was considered to be 1, which was used as control.

Cytokine detection kit based on sandwich ELISA was used for quantitative analysis of IL-6 from macrophages and INF-γ from mice according to instructions. The C57BL/6 mice were intravenously injected with *EGDeABdd-dat* or *EGDeABdd-dat-ompW* of 5 × 10^7^ CFUs, and PBS was the control. On the third day after immunization, mouse serum was collected as antigen for sandwich ELISA.

#### CD4^+^ and CD8^+^ T-Cell Detection

Before T-cell detection, the mice were immunized for three times; 5 × 10^7^ CFUs of *EGDeABdd-dat* and *EGDeABdd-dat-ompW* were intravenously injected into mice (Yang et al., 2014) at 0, 7, and 14 days. PBS with equal volume was the control. One week after the third immunization, the splenocytes were harvested from the mice and stained by the fluorescence-conjugated anti-mouse antibodies CD3-PerCP-Cy5.5, CD4-FITC, and CD8-PE for 15 min. Before detection, unstained and single-stained CD3, CD4, and CD8 cells were used to set machine parameters and compensation. Then, the lymphocyte population is gated according to the cell size on the scatter plot. Finally, cells simultaneously stained with three antibodies flowed through the detector and are displayed on the CD4/CD8 scatter plot.

#### Mouse Immunization and *Aeromonas hydrophila* Challenge

**FIGURE 3:**
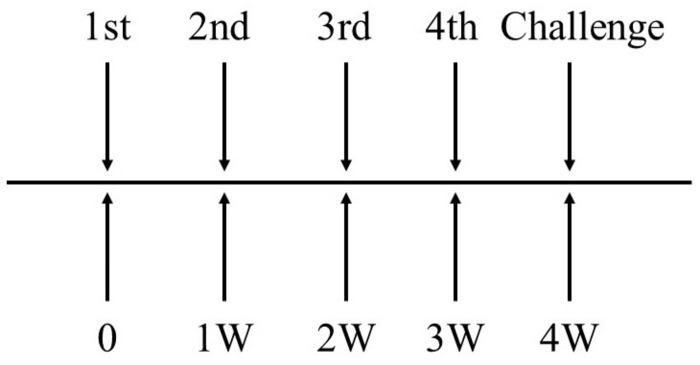
The schematic diagram of immunization plan to mice. Immune dose of *EGDeABdd-dat-ompW* or *EGDeABdd-dat* strain was 5 × 10^7^ CFUs (about 0.1 × LD_50_), and immune interval was 1 week. The mice were immunized four times. One week after the fourth immunization, mice were challenged with AH or *EGDe* of 20 × LD_50_.

The mice were immunized by intravenous injection with *EGDeABdd-dat-ompW* or *EGDeABdd-dat* strain of 5 × 10^7^ CFUs (0.1 × LD_50_), and PBS was the blank control. One week after the fourth immunization, the blood of three mice in each group was collected to detect the antibody produced against AH using the indirect ELISA, and other mice were challenged with AH of 2 × 10^6^ CFUs (20 × LD_50_, LD_50_ about 10^5^ CFUs). The immune interval was 1 week, and the immunization plan is shown in Figure 3.

The same experiment was performed, and the mice were challenged with LM *EGDe* of 2 × 10^7^ CFUs (20 × LD_50_).

### Statistical Analysis

Results of each experiment are analyzed by GraphPad Prism 5 software (San Diego, CA, United States) to compare the differences between groups by one-way analysis of variance followed by an unpaired *t*-test. Values of *P* < 0.05,*P* < 0.01, or *P* < 0.001 were considered significant (^∗^), highly significant (^∗∗^), or extremely significant (^∗∗∗^). Data represent means ± standard deviation (SD).

## Results

### Strain Construction and Analysis

#### Strain Construction of *EGDeABdd-t*

**FIGURE 4:**
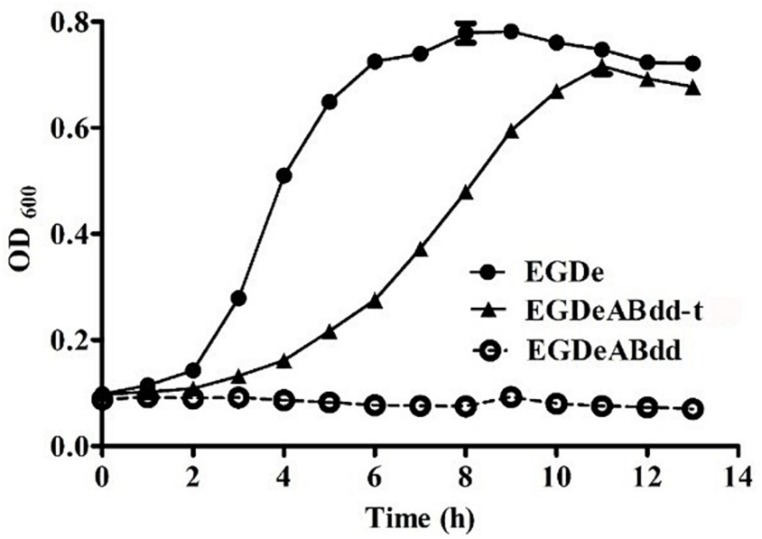
Growth curve of bacteria in normal brain heart infusion (BHI) medium. *EDGe* was wild-type *Listeria monocytogenes* (LM). *EDGeABdd* was a mutant in which the *act*A, *inl*B, *dal*, and *dat* genes were deleted. *EDGeABdd-t* was constructed from *EDGeABdd* containing a pERL3-dat plasmid. *EDGeABdd-t* could survive in normal BHI without D-alanine.

The growth curve of plasmid-supplemented strain was determined by microplate reader at 600 nm every 1 h, and the results are shown in Figure 4. The nutrient mutant *EGDeABdd* could not survive in normal medium, whereas the plasmid-supplemented strain *EGDeABdd-t* restored the abilities of growth and reproduction without exogenous addition of D-alanine. The results indicated the gene *dat* in plasmid successfully expressed the D-alanine transaminase protein to supplement the D-alanine and regulated bacterial growth.

#### Protein Expression of the Vaccine Strain *EGDeABdd-dat-ompW*

**FIGURE 5:**
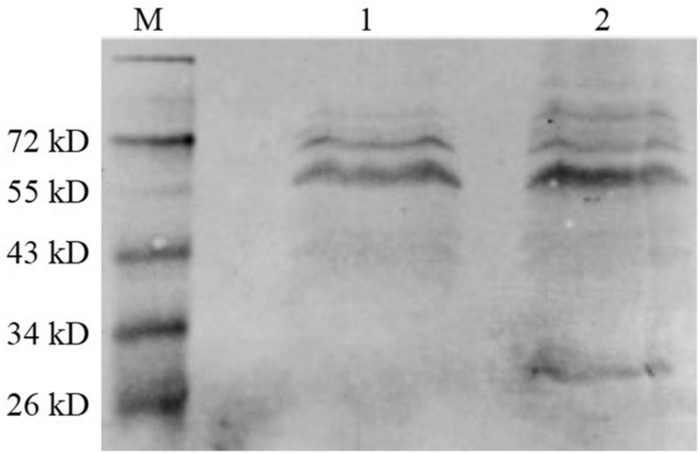
Western blot analysis of exogenous OMPW expression. M was the marker. (1) The control strain *EGDeABdd-dat* containing recombinant plasmid pERL3-dat-tLLO. (2) The vaccine strain *EGDeABdd-dat-ompW* containing recombinant plasmid pERL3-dat-ompW. The fusion protein containing 204 AA of OMPW, 45 AA of tLLO, and 6 His-tag has about 28 kDa. Proteins in culture supernatant were collected using cold ethanol precipitation.

The BHI supernatant of the vaccine strain was collected after overnight culture, and the protein in the supernatant was precipitated using pre-cooled ethanol. The western blot method was used to detect the level of protein expression. As shown in Figure 5, the fusion protein (tLLO-OMPW) was observed in about 28 kDa (lane 2), which was consistent with estimated size. The control *EGDeABdd-dat* has no observed band (lane 1). The results indicated the exogenous gene *omp*W was successfully expressed and secreted to the supernatant of the medium.

#### Gene Expression Detection in Transcription Levels

**FIGURE 6:**
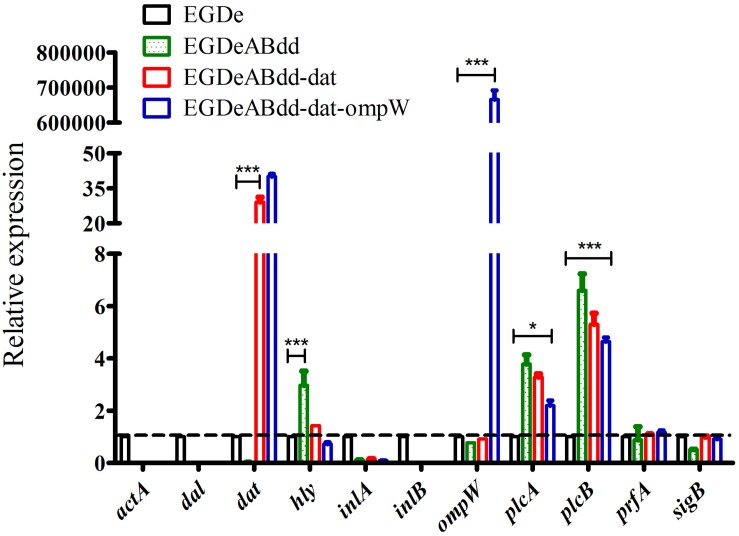
RT-PCR detection of virulence gene relative expression levels. Gene transcription of *EGDe* was considered to be 1, and others were compared with it. Value > 1 indicated gene high expression; otherwise, it represented low expression. *P < 0.05; ***P < 0.001.

The expression levels of virulence gene were detected by qRT-PCR (Ma’ayeh et al., 2018). As shown in Figure 6, the genes including *inl*A, *prf*A, and *sig*B had no significant difference between four strains, whereas the *plc*A and *plc*B genes were significantly increased (value > 1) in strains of *EGDeABdd*, *EGDeABdd-dat*, and *EGDeABdd-dat-ompW* compared with *EGDe*. Meanwhile, the gene *hly* in *EGDeABdd* was significantly increased compared with the other three strains. The gene *dat* in *EGDeABdd-dat* and *EGDeABdd-dat-ompW* and the gene *omp*W in *EGDeABdd-dat-ompW* had a high expression. No detection signals were observed in the *act*A, *inl*B, *dal*, and *dat* genes of *EGDeABdd*, which was further indicated that these genes were successfully deleted.

### Safety Assessment

#### Cell Infection and Induced Caco-2 Apoptosis

**FIGURE 7:**
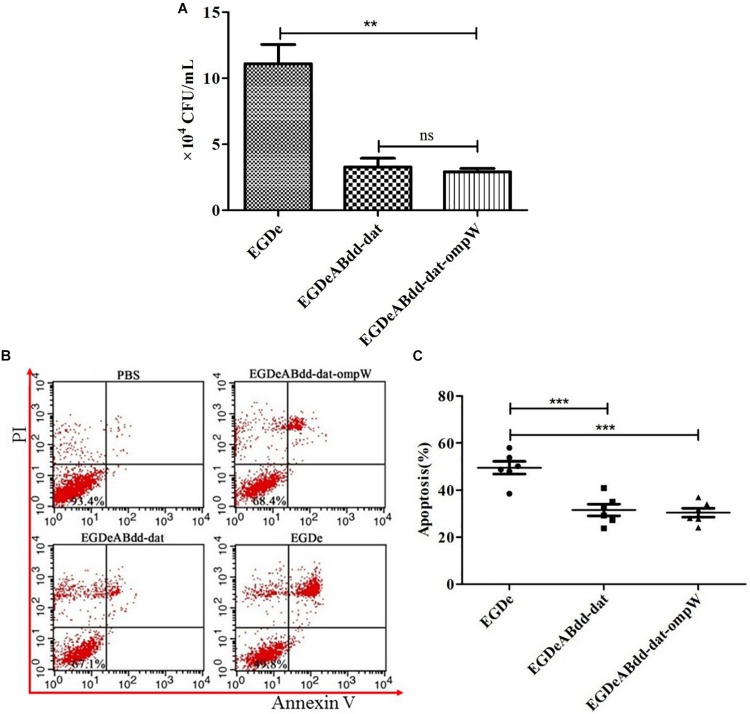
Cytotoxicity of *EGDe*, *EGDeABdd-dat*, and *EGDeABdd-dat-ompW* to Caco-2 cells. **(A)** Number of bacteria invading into cells. The Caco-2 cells were cultured in 12-well plates at 1.0 × 10^5^ cells/well and infected with *EGDe*, *EGDeABdd-dat*, and *EGDeABdd-dat-ompW* at multiplicity of infection (MOI) = 1:100. The infected cells were lysed in 1% Triton X-100 and diluted. The dilutions were counted in brain heart infusion (BHI) plates. **(B)** Impact of *EGDe*, *EGDeABdd-dat*, and *EGDeABdd-dat-ompW* on apoptosis of Caco-2 cells assessed by flow cytometry. Caco-2 cells were invaded by strains for 3 h. The cells were stained with propidium iodide (PI) and fluorescein isothiocyanate (FITC)-conjugated annexin V for 15 min. **(C)** The rates of apoptosis of *EGDe*, *EGDeABdd-dat*, and *EGDeABdd-dat-ompW* to Caco-2 cells. *EGDeABdd-dat* and *EGDeABdd-dat-ompW* had a lower rate of apoptosis than *EGDe*. **P < 0.01; ***P < 0.001.

Caco-2 is colorectal cancer cell line and usually used to simulate the bacterial infection ability of penetrating the intestinal barrier. After incubation with *EGDe*, *EGDeABdd-dat*, and *EGDeABdd-dat-ompW* at MOI = 1:100 for 2 h, extracellular bacteria were killed by penicillin, and then the intracellular bacteria were obtained by plate counting. As shown in Figure 7A, the invasion ability to cells of the vaccine strain *EGDeABdd-dat-ompW* decreased significantly compared with *EGDe*. Meanwhile, results indicated that introducing the *omp*W gene could not cause changes in toxicity, especially in an increase in virulence to cells than could *EGDeABdd-dat*.

Annexin V-FITC/PI apoptosis detection kit and flow cytometry were used for cell staining and signal detection, respectively. From Figure 7B, living cells, early apoptotic cells, late apoptotic cells, and necrotic cells were distributed in annexin V−/PI−, annexin V+/PI−, annexin V+/PI+, and annexin V-/PI + regions, respectively. Results of apoptosis are shown in Figure 7C. From Figures 7B,C, the trend of bacteria inducing Caco-2 cell apoptosis was consistent with infection ability to cells. All the results indicated that the vaccine strain with low cytotoxicity was much safer than *EGDe*.

#### *In vivo* Infection

**FIGURE 8:**
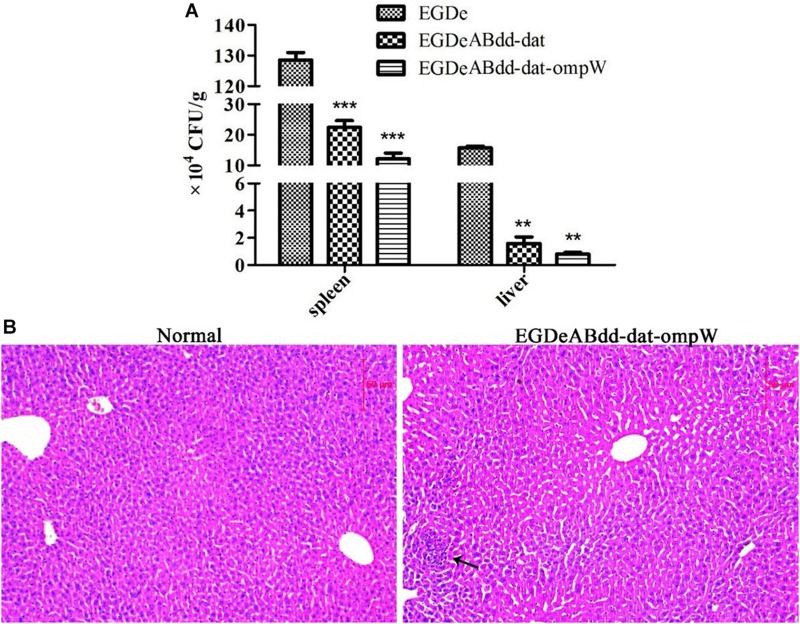
Safety evaluation of vaccine strain to mice. **(A)** Organ distribution of *EGDeABdd-dat* and *EGDeABdd-dat-ompW*. Strains [2 × 10^7^ colony-forming units (CFUs)] were intravenously administered to mice, and CFUs per gram in the spleen and liver were counted on the first day after injection. **(B)** Histological morphology of liver from mice immunized by *EGDeABdd-dat-ompW*. Immunized mice were sacrificed 3 days after intravenous injection of 5.0 × 10^7^ CFUs *EGDeABdd-dat-ompW*. The liver was collected for H&E staining. Slight inflammatory responses were observed in some hepatocytes, and no tissue necrosis or structural damage was seen. **P < 0.01; ***P < 0.001.

After invasion by bacteria for 24 h, the spleen and liver of mice were harvested, and the CFUs per gram were obtained as shown in Figure 8A. The *EGDeABdd-dat* and *EGDeABdd-dat-ompW* strains had significantly lower distribution levels in the spleen and liver, which further indicated that the virulence of the vaccine strain was significantly weakened. Liver section and H&E staining results are shown in Figure 8B, there was only a very mild inflammatory response, and no tissue damage appeared.

### Immunization Evaluation

#### Cytokine Analysis

**FIGURE 9:**
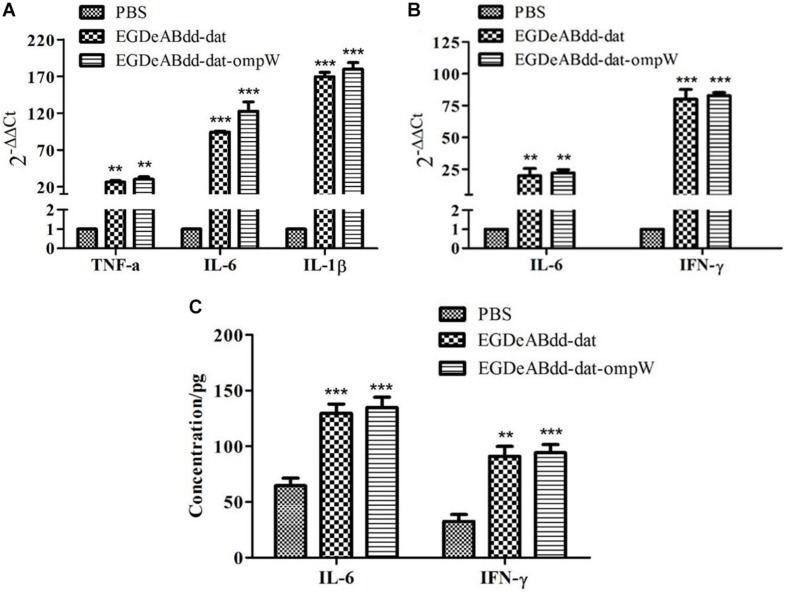
Cytokine analysis of macrophages Raw264.7 and mouse splenocytes by RT-PCR and ELISA. **(A)** Cytokines of macrophages detected by RT-PCR. The multiplicity of infection (MOI) of bacteria and cells was 100:1, and incubation time was 3 h. **(B)** Cytokines of mouse splenocytes detected by RT-PCR. A dose of 5 × 10^7^ colony-forming units (CFUs) of bacteria was injected into C57BL/6 mice, and phosphate-buffered saline (PBS) was the control. On the first day after immunization, the splenocytes were collected for RNA extraction. **(C)** Quantitative analysis of IL-6 from macrophages and IFN-γ from mouse splenocytes using sandwich ELISA. The mice were immunized by 5 × 10^7^ CFUs of bacteria. On the third day after immunization, mouse serum was collected as antigen for sandwich ELISA. **P < 0.01; ***P < 0.001.

Results of cytokines of macrophages Raw264.7 and mice detected by RT-PCR are shown in Figures 9A,B. TNF-α, IL-6, and IL-1β from macrophages had significantly increased after co-culture with strains for 3 h. One day after immunization of mice, IL-6 and IFN-γ of splenocytes had significantly increased. The quantitative analysis of IL-6 of macrophages and IFN-γ of mouse splenocytes using sandwich ELISA are shown in Figure 9C. The concentration of IL-6 and IFN-γ could reach to 90 and 130 pg/ml.

#### T-Cell Immune Responses Analyzed by Flow Cytometry

**FIGURE 10:**
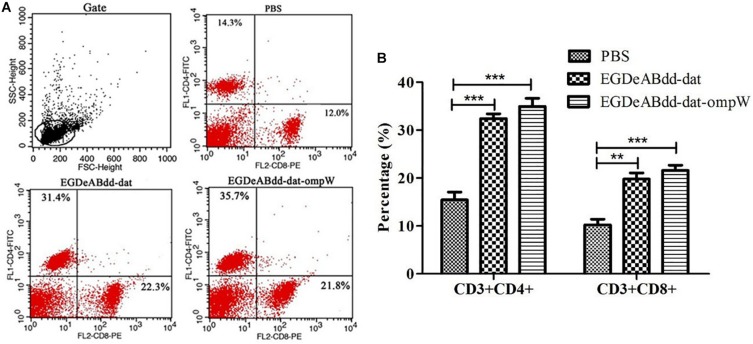
Mouse T-cell immune responses to different bacteria analyzed by flow cytometry. **(A)** Flow cytometry analysis of splenocyte proliferation. One week after immunization with *EGDeABdd-dat* and *EGDeABdd-dat-ompW* for three times using a dose of 5.0 × 10^7^ CFUs (0.1 LD_50_), the splenocytes were collected and stained with CD3, CD4, and CD8 antibodies. **(B)** The percentage of CD4^+^ and CD8^+^ T cells. An increase of T cells appeared in *EGDeABdd-dat* and *EGDeABdd-dat-ompW* groups. **P < 0.01; ***P < 0.001.

Mice’s CD3^+^CD4^+^ and CD3^+^CD8^+^ T-cell immune responses to bacteria were analyzed by flow cytometry. As shown in Figure 10, the percentage of CD4^+^ AND CD8^+^ T cells in *EGDeABdd-dat-ompW* group was significantly increased than those in PBS group, which indicated that the vaccine with low cytotoxicity could induce T-cell immune response to promote T-cell proliferation and activate the MHC class I and MHC class II pathways to present antigens.

#### Mouse Serum Titers and Immune Protection

**FIGURE 11:**
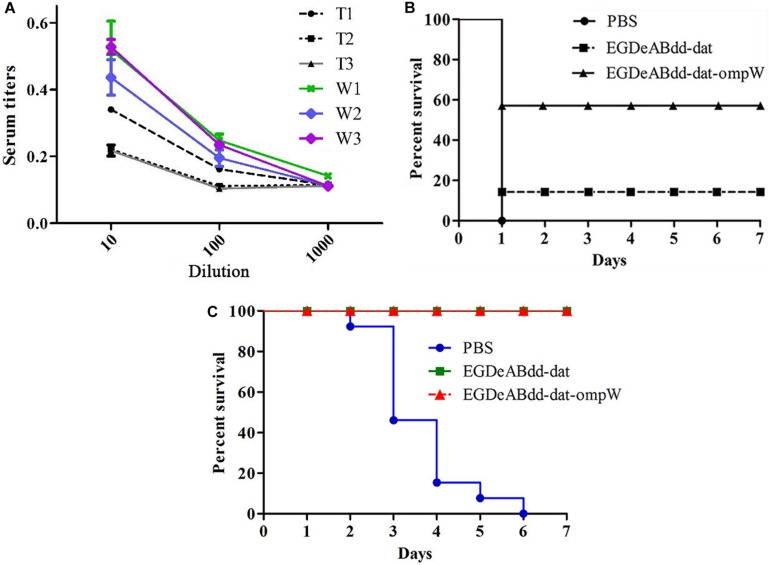
Analysis of humoral immune response and immunoprotective effect. **(A)** Serum titers of mice against *Aeromonas hydrophila* (AH) immunized with *EGDeABdd-dat* and *EGDeABdd-dat-ompW*. T1–T3, mice immunized with *EGDeABdd-dat* for four times using a dose of 5 × 10^7^ colony-forming units (CFUs), and the immune interval was 1 week. W1–W3, mice immunized with *EGDeABdd-dat-ompW* for four times using a dose of 5 × 10^7^ CFUs, and the interval was 1 week. **(B)** Immunoprotective effect of phosphate-buffered saline (PBS), *EGDeABdd-dat*, and *EGDeABdd-dat-ompW* groups to AH. One week after the fourth immunization, serum collection and challenge were performed. The mice in PBS, *EGDeABdd-dat*, and *EGDeABdd-dat-ompW* groups were challenged with AH of 2 × 10^6^ CFUs. **(C)** Mice were challenged with *Listeria monocytogenes* (LM) *EGDe* using a dose of 2 × 10^7^ CFUs. The *EGDeABdd-dat* and *EGDeABdd-dat-ompW* groups could provide a 100% protective effect.

One week after the fourth immunization, the serum titers from different groups were measured by indirect ELISA. Indirect ELISA was performed as follows (Zeng et al., 2016). Gradient serum was added to a 96-well plate coated with antigen (AH) for incubation at 37°C for 1 h. After being washed for three times, horseradish peroxidase (HRP)-conjugated goat anti-mouse IgG antibody was added and reacted for 1 h. After being washed for three times, the TMB substrate was added for 15 min. The reaction was terminated by 2 M of sulfuric acid. Absorbance values were measured at 450 nm. When the values were higher than 2.1 × negative values, the results were judged to be positive, and the maximum dilution of serum was the titer of the mouse. As shown in Figure 11A, serum titers of *EGDeABdd-dat-ompW* group could reach to 1:100, whereas the titers of *EGDeABdd-dat* group were 1:10.

On the same day, the immunized mice in PBS, *EGDeABdd-dat*, and *EGDeABdd-dat-ompW* groups were challenged with AH with a dose of 2 × 10^6^ CFUs (20 × LD_50_), and the results are shown in Figure 11B. All the mice in PBS group died within 2 days, whereas the *EGDeABdd-dat* and *EGDeABdd-dat-ompW* groups could provide an immunoprotective effect of 18 and 58%, respectively, after immunization for four times.

The immunized mice in PBS, *EGDeABdd-dat*, and *EGDeABdd-dat-ompW* groups were also challenged with LM *EGDe* with a dose of 2 × 10^7^ CFUs (20 × LD_50_), and the results are shown in Figure 11C. All the mice in PBS group died within a week, whereas the *EGDeABdd-dat* and *EGDeABdd-dat-ompW* groups could provide a 100% immunoprotective effect.

## Discussion

LM has been shown to have an exceptional capacity to induce anti-tumor immunity to tumor-associated antigens, such as HPV16 E7 (Jia et al., 2017), influenza virus NP3 (Pan et al., 1999), HIV-1 gag (Okuda et al., 1995; Jiang et al., 2007), tuberculosis (Yin et al., 2017), metastatic pancreatic cancer (Le et al., 2016), and canine osteosarcoma (Mason et al., 2016). In this study, an AH vaccine using attenuated LM as delivery vector was described, and the safety and the immunogenicity of the vaccine were assessed. The attenuated LM vector of the *dal*, *dat*, *act*A, and *inl*B gene deletions harbors a plasmid to express antigens, and in this study, the *dat* gene from LM and AH antigen OMPW was successfully implemented. By plasmid supplementation of the *dat* gene in auxotrophic mutant *EGDeABdd*, recombinant plasmid could stably exist in a strain without antibiotic screening. Owing to the extensive distribution in a variety of bacteria, such as LM, *Escherichia coli*, and *Bacillus subtilis*, the *dal* gene was considered more important than the *dat* gene in LM. The construction concept of the carrier was to replenish the *dal* gene of *B. subtilis* in previous studies (Yang et al., 2014), whereas the gene *dat* of LM selected for plasmid carrying was successfully implemented in this study.

The results of *in vivo* and *in vitro* studies provided evidence demonstrating that the vaccine strain had less virulence than had *EGDe*. The results of immunization experiments suggested that the vaccine had the ability to present antigen to the immune system and could provide 58% immunoprotective effect higher than 18% of the vector group. Meanwhile, the vaccine could induce the expansion of CD3^+^CD4^+^ T cells and CD3^+^CD8^+^ T cells; thus, it could induce immune responses to kill cells infected by bacteria. All the results indicated that the *EGDeABdd-dat-ompW* strain used as a vaccine for AH was safe and highly immunogenic.

An ideal LM vector vaccine designed for animal or human use has been achieved, which the bacterium is less virulent yet still retains its ability to present antigen to the immune system. Owing to the reduced virulence and increased safety, the *dal*, *dat*, *act*A, and *inl*B deletion strains were superior to the *dal*/*dat* deletion or *act*A/*inl*B deletion strain as a vaccine vector. In conclusion, the *EGDeABdd-dat-ompW* vaccine could stably secrete the OMPW, an OMP of AH, as a protective antigen to elicit a specific immune response. *EGDeABdd-dat-ompW* strain is a safe and effective vaccine that could stimulate host to produce antibody in serum against AH and improve survival when challenged with AH. The successful application of strain *EGDeABdd-dat* in an AH vaccine, indicating *EGDeABdd-dat* strain as a vaccine vector in future clinical use, will be realized soon. The broader goal of the study is to extend the application of the live attenuated vector to designing potential constructs expressing other single or multiple heterologous antigens.

## Data Availability Statement

All datasets generated for this study are included in the article/supplementary material.

## Ethics Statement

All animal studies have been approved by the China Ethics Committee and performed in accordance with the ethical standards.

## Author Contributions

All authors listed have made a substantial, direct and intellectual contribution to the work, and approved it for publication. HZ was responsible for experimental operation and article writing. MX, CD, and JM gave experimental help. DX, XW, and JQ gave article modification help. QL designed the experiment.

## Conflict of Interest

The authors declare that the research was conducted in the absence of any commercial or financial relationships that could be construed as a potential conflict of interest.
